# A birdstrike risk assessment model and its application at Ordos Airport, China

**DOI:** 10.1038/s41598-020-76275-z

**Published:** 2020-11-12

**Authors:** Yuanyuan Hu, Pu Xing, Fan Yang, Gang Feng, Guisheng Yang, Zhengwang Zhang

**Affiliations:** 1 School of Life Sciences, Inner Mongolia University, Hohhot, 010070 China; 2 The Hohhot Branch of Inner Mongolia Autonomous Region Civil Airport Group Co., LTD, Hohhot, 010070 China; 3 School of Ecology and Environment, Inner Mongolia University, Hohhot, 010021 China; 4 School of Life Sciences, Beijing Normal University, Beijing, 100875 China

**Keywords:** Animal behaviour, Ecological modelling

## Abstract

Birdstrikes are an important threat to aviation safety. A standardized, scientific process for assessing birdstrike risk could prevent accidents, thereby improving the flight safety and reducing economic losses. However, China currently lacks a unified birdstrike risk assessment system. Here, we propose and validate a new model for assessing birdstrike risk in order to fill that need. The model consists of two elements. First, empirical data are collected on the occurrence of birds at the airport and in a surrounding 8 km buffer. Second, each species is evaluated with a risk assessment matrix that takes into account the number of birds, weight, flight altitude, a tendency to cluster, and range of activity. These five factors allow each species to be divided into one of three risk levels: high danger (level 3), moderate danger (level 2) and low danger (level 1). We propose corresponding birdstrike prevention measures for each level. We apply this method to the civil aviation airport in Ordos, China. We found that 20 of the 118 species of birds in and around the airport were high danger birds (level 3). To validate this process, we compared these species with records of birdstrike accidents in a database maintained by the Civil Aviation Administration of China (CAAC) for 2007–2016. We found that 42% of the species we identified as high risk had been involved in at least one birdstrike accident, and that the remaining 58% belonged to families that appeared in the database. The high degree of overlap gives us high confidence in the practicality of our risk assessment model, which is based on the risk management concept of ISO 31000. Critically, this new model and method for predicting bird strike risk can be replicated at other airports around the world, even where no extensive records have been kept of past birdstrikes.

## Introduction

A birdstrike is an incident in which an aircraft collides with a flying bird at an airport or an adjacent area^1^. In recent years, the number of aircraft taking off and landing has increased continuously, and with increasing traffic has come an increasing number of birdstrike events. They are one of the most important threats to aviation safety. According to the statistics of the Civil Aviation Administration of China (CAAC) “Bird Strike Aircraft Information Analysis Report”, birdstrike accidents increased from 326 in 2007 to 4618 in 2016. The number of strikes reported by the Federal Aviation Agency (FAA) in the United States has increased from 1851 in 1990 to a record 13,668 in 2014^2^. The increasing birdstrikes cause aircraft grounding and delays, which not only affect travel arrangements but also incur huge security risks and economic losses^3^. The International Federation of Aviation upgraded birdstrikes to an "A" aviation disaster.

In order to reduce birdstrike accidents as much as possible, scholars have proposed different methods for assessing birdstrike risk. Most countries use a traditional risk assessment matrix to judge risk based on the number of birdstrikes in recent years^4,5^. In the United Kingdom, Allan^4^ used both national and airport-specific data on the degree of damage to aircraft to evaluate bird risk, creating a simple probability-times-severity matrix. A model for China should utilize a similar matrix, however, the CAAC does not have a similarly detailed database of past birdstrike events. So we refer to this matrix, but the data sources used to calibrate the model are different. In Italy, Soldatini et al.^6^ established the Birdstrike Risk Index (BRI)^7^ using an ecological approach. The same authors created a second version (BRI2) incorporating new time scales that allow for appropriate management planning. Coccon used Generalized Linear Models (GLMs) with binomial distributions to estimate the contribution of habitats to wildlife use of the study area, as a function of the season^5^. In China, Ning proposed a real-time birdstrike risk evaluation method based on the bird information collected by the airport-based avian radar system and the flight phase of the aircraft provided by the air-traffic control system^8^. However, most airports in China are not equipped with radar devices, so this method is not universally applicable. Chinese scholars have typically calculated the risk of different types of birdstrikes according to one or a subset of seven factors: comparative space, comparative time, comparative quantity, comparative weight, distance coefficient, cluster coefficient and flight height. However, many of these criteria are subjective or cannot be made in a consistent way across different airports^9–13^. The lack of adequate birdstrike data at Chinese airports means a statistical analysis using existing methods would be inappropriate.

In this study we attempt to address the lack of a universally applicable birdstrike risk assessment process that can be used in countries and settings were past birdstrike data is incomplete or absent. Our model is based on assessing five readily measured bird species traits: relative quantity component, relative weight component, flight height, cluster coefficient, activity range risk coefficient. We then collected relevant bird data from Ordos Airport and its vicinity, scoring species by the risk criteria. We attempt to validate our model by assessing whether the birds in our highest risk category have indeed been associated with past birdstrike events in China. This model attempts to standardize a birdstrike risk prevention system, by being applicable to birdstrike prevention work in airports all around the world.

## Results

### Bird species and risk classification at Ordos Civil Aviation Airport

We recorded 118 bird species representing 14 orders and 36 families.

According to our birdstrike risk assessment model, the birds at the Ordos Civil Aviation Airport and in the surrounding 8 km buffer are classified into three levels: high danger (level 3), moderate danger (level 2) and low danger (level 1) (Supplementary Table 1).

We found that 20 species at the airport met the criteria for high danger birds (level 3), accounting for 17.0% of the total number of bird species in the airport. We found that 32 species met the criteria for moderate danger birds, accounting for 27.1% of the total bird species. The remaining 66 species were classified as low danger birds, accounting for 55.9% of the total number of bird species.

**Figure 1:**
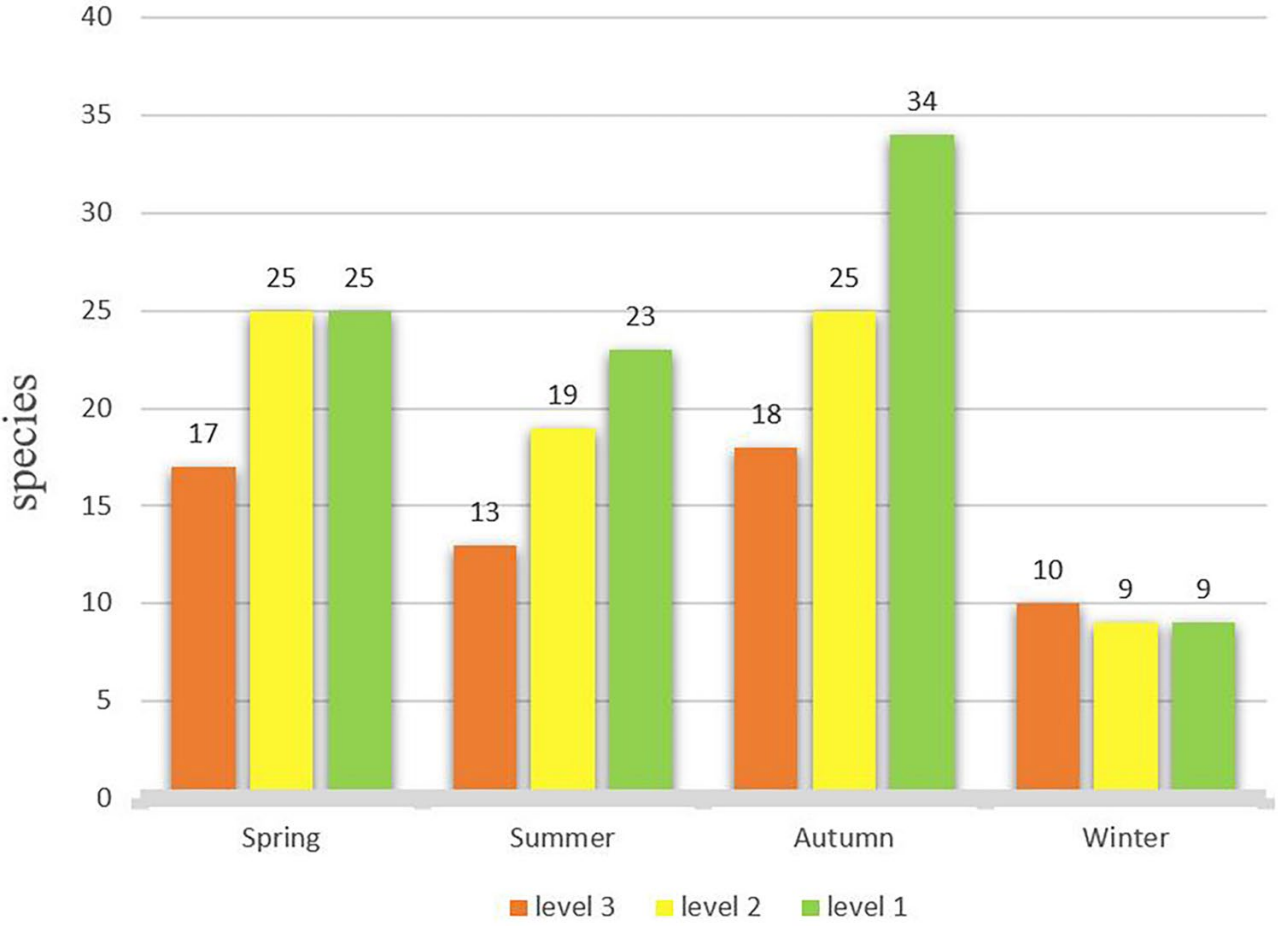
A bar graph comparing the number of bird species at Ordos Airport, China in three different birdstrike risk classes during each season.

Of the 20 species of high danger birds, 18 species were present in the autumn, 17 in spring, 13 in summer and just 10 in winter. In spring and autumn, we encountered the greatest number of moderate danger bird species, 25 during each season (Fig. 1).

### Feasibility analysis of birdstrike risk assessment at Ordos Airport

In order to test the feasibility of our birdstrike risk assessment model, we counted the species identities of birds that caused birdstrike accidents between 2007–2016 according to a record of events maintained by CAAC (https://www.birdstrike.cn/). The species of birds that caused birdstrike events were rock pigeon, ring-necked pheasant, cattle egret, black-crowned night heron, ruddy shelduck, black kite, short-eared owl, common kestrel, tree sparrow, oriental turtle dove, house swift and eagle. Of these species, four were among the species at Ordos Airport that we determined to be in the highest danger category (pigeons, ring-necked pheasant, ruddy shelduck, and common kestrel). Other bird species at the Ordos that we assessed to be Level 3 risks belonged to the families ardeidae, accipitridae, falconidae, columbidae, and passeridae, all of which have also appeared on the list of birdstrike accidents in China Civil Aviation 2007–2016.

## Discussion

Birds at the airport and in the surrounding areas are potentially dangerous to aircraft flight safety, but the risk is not uniform across all species. The 20 species high danger birds surveyed at the Ordos Airport should be the focus of prevention efforts. The airport should pay attention to strengthening prevention in the spring and autumn. The nature of these prevention measures depends on the ecology characteristics of the high risk species.

The waterfowl in our study area include the swan goose, bean goose, tundra swan, whooper swan, ruddy shelduck, common teal and red-crested pochard in anseriformes, the little grebe in podicipediformes, the coot and demoiselle crane in gruiformes, and the white spoonbill in pelecaniformes. Among them, the swan goose, bean goose, tundra swan, whooper swan, and common teal are passing migrant birds, and the rest are summer resident birds. The Honghaizi Wetland, which is 5.59 km away from the Ordos Airport, is an important habitat of these waterfowl. Because these birds are relatively large in size and fly in clusters, they are more damaging when involved in birdstrikes. Airports can avoid bird strikes by adjusting flight schedules during the morning and evening peak times for birds foraging and migration.

High risk terrestrial birds include the ring-necked pheasant in galliformes and the rock pigeon and Eurasian collared dove in columbiformes. The ring-necked pheasant, which is a common seed-feeding resident bird, has a large size, fast flight speed but does not have a long single flight distance. The flying height of Ring-necked pheasant is usually below 10 m, meaning they are only a risk when they are present on runways. The species sometimes does cross the tarmac of airports, thus, the airport’s manager should control the grass height and extent before a large number of seeds are mature in the autumn, thereby destroying the nesting and feeding conditions for these birds^14^. Rock pigeons are often found in fast-moving clustered colonies, which means they can be a great threat to aircraft flight safety. Other than controlling the food source of the species, the airport’s manager should also coordinate with the local government to restrict pigeon rearing in the surrounding area. If necessary, these birds can be killed or scared off with gunfire.

Raptors include the steppe eagle in accipitriformes and the common kestrel, merlin, and Eurasian hobby in falconiformes. The raptors are heavy and their flight altitude is similar to the height of the aircraft when it takes off or lands. Moreover, the flight speed of the raptors is fast. These raptors are often seen hovering over airports. Therefore, the raptors are a serious threat to flight safety when they appear close to an aircraft’s flight path. This risk can be mitigated by reducing the food resources for the raptors around airports, including timely clearing of the birds stuck to bird nets. Rodenticides should be regularly placed at the airport, especially before the peak of rodent breeding and in the winter when food is scarce.

Songbirds include the common magpie and tree sparrow in passeriformes. Common magpies and tree sparrows are omnivorous birds that can be found around various environments throughout the year. They often gather in large groups in the early morning and evening, causing the safety hazards for aircraft. To reduce their food resource availability, grass should be cut below 20 cm and bugs should be eliminated^15^, and the area of open water at the airport should be reduced and drainages should be covered^16^. In addition, it is necessary to regularly clean up garbage at the airport and in the surrounding residential areas in time and regularly remove the birds’ nests from the high trees and facilities around the airport. The common magpie has strong adaptability to airport bird repellent equipment, therefore, regularly changing the intimidation techniques is required. In case of a serious threat, they can be shot with a shotgun.

At present, the data on the birdstrikes at airports urgently needs to be improved. If actual birdstrike data is used as the criterion for the likelihood and severity of birdstrikes, our generic process could become more targeted and effective, particularly if very recent data could be used to assess trends and forecast year-on-year changes in risk. In the future, when assessing the year's dangerous bird species, officials could consult the actual birdstrike data for the previous three years as an additional criterion for evaluation. It can also be combined with the degree of aircraft damage.

Individual bird species hazardous levels could also be adjusted based on existing data: if a species is involved in birdstrikes at an airport three times in the last three years, that species’ hazard level could be raised one level. Similarly, if in the non-responsible area of an airport, birdstrikes of the same species account for more than 10% of the total number of bird strikes, then the danger rating could be raised one level.

According to the birdstrike report by CAAC, the species that caused a strike is generally unknown. From 2007 to 2016, a total of 21,599 birdstrikes occurred, but the species causing the strike was only confirmed in 838 cases, or 3.9%. In only two years of the decade-long dataset was the proportion greater than 5%. For six years it was less than 3%.

The data collected on the bird species and the height of birdstrikes at airports in China are seriously lacking. Therefore, if there are bird corpses, blood stains and feathers that are difficult to be identified, they should be sent to scientific research institutions for DNA identification. We recommend that relevant state agencies appropriately adjust the evaluation methods of airport birdstrikes in order to improve our understanding of the risk posed by different bird species. There is an urgent need to form an analyzable database for birdstrike events. In the current era of big data, such a database would provide valuable information needed for the targeted prevention of birdstrikes.

This paper combines birdstrike risk assessment methods developed in other countries with empirical data from a Chinese airport. In doing so, it puts forward a complete birdstrike risk assessment model, providing a replicable process for how to survey birds at an airport and its surroundings and how to determine the risk each species poses so that airports can take concrete measures to reduce the number of dangerous birds, and thereby reduce the risk of birdstrike. In addition, this model also provides a blueprint to facilitate long-term, dynamic evaluations of birdstrike prevention efforts at airports. In other words, our model is not static, after an airport initially applies this process, the birdstrike risk assessment mechanism still needs further research and improvement using new empirical data.

## Methods

### Study area and data survey method

Ordos Civil Aviation Airport, our study site, is located in the southwestern part of the Inner Mongolia Autonomous Region, China. It is characterized by mid-temperate continental climate and is located on the northeastern edge of the Mu Us Desert. Its main characteristics are a long winter and short summer, but has four distinct seasons. The mean annual temperature is 6.2 ℃, and the mean annual precipitation is 358 mm, mostly concentrated between June and August. The mean annual wind speed is 3.6 m/s^17^. There are five main land cover types in Ordos civil aviation airport and its surrounding areas: farmland, residential areas, woodland, shrub grassland and wetland.

We used the line transects method and point counts method to investigate the environment and birds within our study area. It is a commonly used bird survey method, and is also widely used in the survey of birds in and around airports. The line transects were 1000 m × 100 m and the walking speed was 1.5–2.0 km/h. We observed and recorded birds with 10 × 50BA and 30 × 77BA Leica telescopes, SLR digital cameras (Canon 5D Mark III) with telephoto lenses (Canon 100–400 mm). The point count method we used had an observation radius of 200 m. We observed birds with 10 × 50BA Leica binoculars and a 30 × 77BA Leica fixed-mount spotting scope^18–22^. The flight altitude was estimated using a visual comparison method: an altimeter was used to measure the height of trees and buildings in the observation area and these heights were used to estimate the flight altitude of observed birds. Bird identifications were based on *A Field Guide to the Birds of China*^23^.

The survey areas were divided into three areas: A, B and C. Section A was located within the boundary of the airport. Section A surveys consisted of five shrub grassland transects, with one transect on the runway and one on the apron. Section B was the area within 4 km of the center of the airport (but excluding Section A). There were five woodlands, nine shrub grassland, two farmland and four residential areas. Section C consisted of all areas located with 8 km of the center of the airport, excluding Sections A and B. In Section C we established three woodlands transects, three shrub-grassland transects, two farmland transects and four wetland transects. The species, quantity, distribution, cluster and flight altitude of birds in 39 transects or point counts set up within 8 km of the airport and its surrounding areas were investigated monthly by the method of sample strip or sample point (wetland using sample point method). A total of 468 individual point count or transect surveys were conducted over the study year.

### Birdstrike risk assessment model

**Figure 2:**
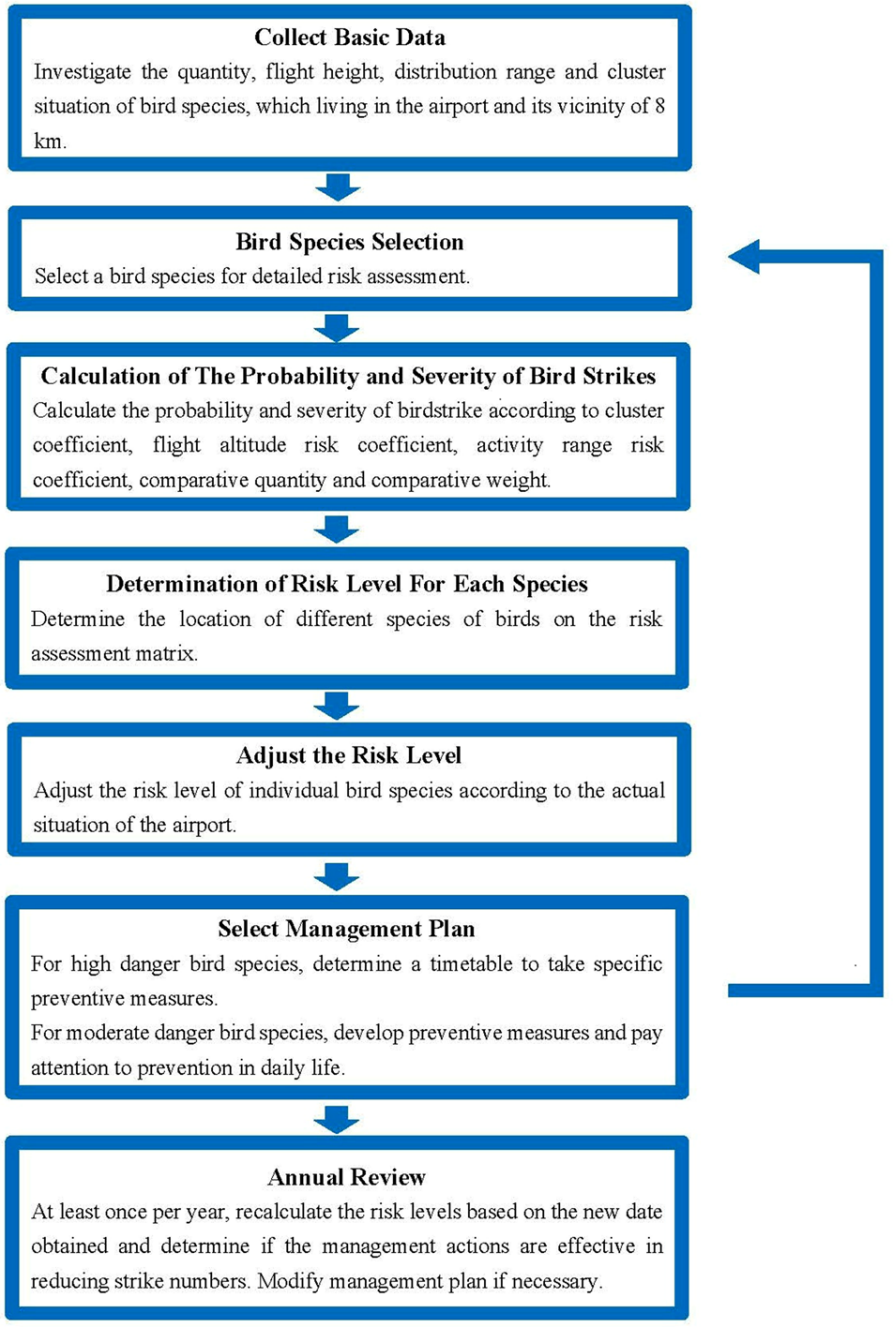
Flow chart of the airport birdstrike risk assessment process.

Investigating the bird situation in the airport and surrounding areas is a prerequisite for birdstrike prevention. The establishment of a scientific and standardized risk assessment process for birdstrike prevention (Fig. 2) is helpful for the systematic evaluation of birdstrike risk. This model is based on the ISO 31000 risk management process^24^—risk identification, risk analysis, risk assessment, risk response, risk recording and reporting, communication and consultation, monitoring and review. A flow chart for bird strike risk assessment was constructed.

The occurrence of a birdstrike is a matter of probability. The consequences of a birdstrike are a matter of severity, with loss of aircraft or life occurring in extreme cases. Together they combine to determine birdstrike risk, and thus our five risk factors are meant to capture severity and likelihood. The first risk factor is the comparative number, which is important for the simple reason that if a bird species collides with an airplane, a greater number of birds have more serious consequences for an airplane. Among the bird strike events between 2007 and 2014 with the largest record impact energy, half of them involved species in the family Anatidae, and they were all birds with a relatively large comparative number^25,26^. The second risk factor is bird weight. The greater the weight of a bird, the greater the force generated by an aircraft impact, and the severity of birdstrikes will also increase. Flight altitude is an important factor in the analysis of birdstrike risk^12^. According to ICAO data, we use 40 m as the critical value of the risk zone. If the average flight height of a bird species is closer to the critical value, the risk of birdstrike will be higher^12^. Our fourth risk factor is a clustering coefficient, which relates to the living habits of a bird species to move in large groups. If a bird species often gather in large numbers, then the possibility of encountering an aircraft and causing a birdstrike event is greater. This is due to the nature of the collective behavior of birds while flying in flocks of murmurations. Following large, tight formations, birds make fewer independent moving decisions, being forced to constantly react to the movements of their neighbors and having their view partially obscured. They may not have space to avoid oncoming aircraft, or may lack the freedom and alert to choose a successful escape path leading to a higher probability of collision with the aircraft^27,28^. About 80% of birdstrikes occur during the take-off, climb, approach, and landing phases of flights^12,13,29,30^, so the distance between bird activity from the flight zone is also an important factor in assessing the probability of birdstrikes. Combining with the above analyses, a risk assessment matrix based on the five factors of bird number, weight, flight altitude, cluster coefficient and range of activity was proposed to assess the risk level of bird species in the airport and its surrounding area within 8 km.

#### Risk factor assignment

1. Comparative number = (the number of individual birds/the number of individuals with the most number of birds) × 100.
2. Comparative weight = (estimated weight of all birds of a single species/the largest weight of all birds of any species) × 100.
3. Risk coefficient of flight height:

**Table:** 

Flight height H (m)	Risk coefficient of flight height
H > 100	0.1
100 ≥ H > 50	0.5
50 ≥ H > 30	1
30 ≥ H > 5	0.5
5 ≥ H	0.1
4. Clustering coefficient assignment:

**Table:** 

Number of individuals of a cluster	Cluster coefficient
N > 100	1
100 > N ≥ 20	0.5
20 > N ≥ 3	0.2
3 > N ≥ 1	0
5. Activity range risk coefficient assignment: according to the bird species observed area, it could be divided into three levels: activities in flight area, activities within 4 km from flight area, activities within 8 km from flight area but not within 4 km. If a bird species has activity in each area, the nearest one to the flight zone will be used as the input for the risk assessment model. The birds distributed in these three regions were assigned 0.9, 0.6 and 0.3 respectively.

#### Risk assessment matrix

$$ {\text{Likelihood }} = \, \left( {{\text{cluster coefficient }} + {\text{ Risk coefficient of flight height }} + {\text{ Activity range risk coefficient}}} \right) \, \times { 1}00 \, /{ 3} $$

$$ {\text{Severity }} = \, \left( {{\text{comparative number }} + {\text{ comparative weight}}} \right) \, \times { 1}00/{2} $$

The expert evaluation method is used to determine the numerical range^31^ (Table 1).

**Table 1:** Birdstrike likelihood and severity rating.

Likelihood	0–14	15–29	30–49	50–69	70–100
	Very low	Low	Moderate	High	Very high
Severity	0–3	4–6	7–13	14–39	40–100
	Very low	Low	Moderate	High	Very high

According to the very low, low, moderate, high and very high levels of possibility and severity (Table 1), the level of potential threatening birds are divided into three risk levels: high danger (level 3), moderate danger (level 2), and low danger (level 1). (Table 2).

**Table 2:**
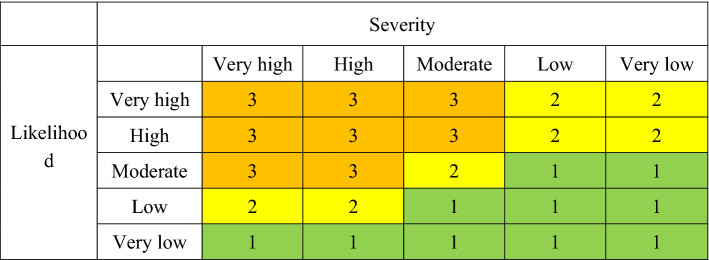
Airport birdstrike risk assessment matrix.

Adjust the risk level of individual bird species according to the actual situation of the airport:

1. If the bird is a raptor, increase the risk level by one.
2. The risk level for bird species that are seen crossing a runway or passing through the sky above the runway more than three times should be increased by one.

Raptors fly fast, and collisions with airplanes can have very serious consequences. Among the birdstrike events with the largest record of birdstrike impact energy from 2007 to 2014, half of them were raptors. However, because their weight is actually low compared to birds like ducks, and their solitary habits, the risk level calculated by this method is often lower than the actual risk, so the risk level of the raptor is increased by one level. Most birdstrikes occur when the aircraft takes off and lands. If the bird's movement often crosses the runway or the nearby sky, it is more likely to cross an aircraft’s flight trajectory, and therefore is very dangerous for the aircraft. For this reason, when a bird species is seen crossing the runway and flying over the top of the runway three times, the risk level of that species should be increased by one.

Each airport should adjust their assessments based on locally collected empirical data on strike likelihood and severity as well as ongoing bird monitoring at the airport and its surrounding environment.

## Supplementary information

Supplementary Information.

## Data Availability

All data generated or analysed during this study is available for this paper and supplementary information.
