# Supplementary material for: A birdstrike risk assessment model and its application at Ordos Airport, China
Source: Sci Rep. 2020 Nov 12;10:19627. doi: 10.1038/s41598-020-76275-z (PMC7661541; doi:10.1038/s41598-020-76275-z)
Supplement: Supplementary file 1 — Supplementary Information. [file 41598_2020_76275_MOESM1_ESM.pdf]

A Birdstrike Risk Assessment Model and Its Application at Ordos Airport, China

Yuanyuan Hu[1], Pu Xing[2], Fan Yang[1], Gang Feng[3], Guisheng Yang[1*], Zhengwang Zhang[4]

[1]School of Life Sciences, Inner Mongolia University, Hohhot, 010070, China

[2]The Hohhot Branch of Inner Mongolia Autonomous Region Civil Airport Group co., LTD, 010070, China

[3]School of Ecology and Environment, Inner Mongolia University, Hohhot, 010021, China

[4]School of Life Sciences, Beijing Normal University, Beijing, 100875, China

Supplementary table 1 Bird composition and risk assessment at Ordos airport

specific name	habitat distribution	Resident type	comparative number	comparative weight	Risk coefficient of flight height	Cluster coefficient	Activity range risk coefficient	Possibility（%）	Severity（%）	risk level
Rock Partridge *Alectoris chukar*	ST	R	0.0065	0.0600	0.1	0.2	0.9	40.00	3.32	1
Daurian Partridge *Perdix dauurica*	STF	R	0.0247	0.0296	0.1	0.2	0.9	40.00	2.71	1
Ring-necked Pheasant *Phasianus colchicus*	STF	R	0.0092	0.1534	0.1	0.5	0.9	50.00	8.13	3
Swan Goose *Anser cygnoides*	W	P	0.0567	0.3737	0.5	1	0.3	60.00	21.52	3
Bean Goose *Anser fabalis*	W	P	0.0843	0.3316	0.5	1	0.3	60.00	20.79	3
Tundra Swan *Cygnus columbianus*	W	P	0.0604	0.5868	0.5	1	0.3	60.00	32.36	3
Whooper Swan *Cygnus cygnus*	W	P	0.1972	1.0000	0.5	1	0.3	60.00	59.86	3
Common Shelduck *Tadorna tadorna*	W	S	0.0002	0.1184	1.0	0	0.3	43.33	5.93	1
Ruddy Shelduck *Tadorna ferruginea*	W	S	0.2352	0.1398	1.0	1	0.3	76.67	18.75	3
Gadwall *Anas strepera*	W	S	0.0227	0.0934	1.0	0.5	0.3	60.00	5.80	2
Wigeon *Anas penelope*	W	P	0.0002	0.0763	1.0	0	0.3	43.33	3.83	1
Mallard *Anas platyrhynchos*	W	S	0.0274	0.1211	0.5	0.5	0.3	43.33	7.42	2

Spot-billed Duck *Anas poecilorhyncha*	W	S	0.0057	0.1195	0.5	0.5	0.3	43.33	6.26	2
Northern pintail *Anas acuta*	W	P	0.0002	0.09	0.5	0	0.3	26.67	4.51	1
Common Teal *Anas crecca*	W	P	0.7824	0.0308	0.5	1	0.3	60.00	40.66	3
Shoveller *Anas clypeata*	W	S	0.0058	0.0579	0.5	0.5	0.3	43.33	3.18	1
Red-crested Pochard *Netta rufina*	W	S	0.0144	0.1157	1.0	1	0.3	76.67	6.50	3
Common Pochardl *Aythya ferina*	W	S	0.0079	0.0912	1.0	0.5	0.3	60.00	4.95	2
Ferruginous Pochard *Aythya nyroca*	W	S	0.0253	0.0658	1.0	0.5	0.3	60.00	4.55	2
Tufted duck *Aythya fuligula*	W	P	0.0097	0.0692	1.0	0.5	0.3	60.00	3.94	2
Greater Scaup *Aythya marila*	W	P	0.0002	0.0963	1.0	0	0.3	43.33	4.83	1
Little Grebe *Tachybaptus ruficollis*	W	S	0.0143	0.2184	0.5	0.5	0.3	43.33	11.64	3
Great Crested Grebe *Podiceps cristatus*	W	S	0.0107	0.0868	0.5	0.5	0.3	43.33	4.88	1
Horned Grebe *Podiceps auritus*	W	P	0.0007	0.0016	0.5	0.2	0.3	33.33	0.11	1
Black-necked Grebe *Podiceps nigricollis*	W	S	0.0013	0.0311	0.1	0.2	0.3	20.00	1.62	1
Rock Pigeon *Columba sp.*	A	R	0.1105	0.0299	1.0	1	0.9	96.67	7.02	3
Eurasian Collared Dove *Streptopelia decaocto*	A	R	0.1046	0.0195	0.5	1	0.9	80.00	6.20	3
Spotted Dove *Streptopelia chinensis*	STFN	R	0.0017	0.0168	0.5	0.2	0.9	53.33	0.92	2
Common swift *Apus apus*	W	S	0.0002	0.0049	1.0	0	0.3	43.33	0.26	1
Commom Cuckoo *Cuculus canorus*	STW	S	0.0019	0.0133	0.5	0.2	0.6	43.33	0.76	1
Coot *Fulica atra*	W	S	0.5494	0.0713	0.1	1	0.3	46.67	31.04	3

Demoiselle Crane *Anthropoides virgo*	W	S	0.0001	0.2513	1.0	0	0.3	43.33	12.57	3
Black-winged Stilt *Himantopus himantopus*	W	S	0.0240	0.0193	0.5	0.5	0.3	43.33	2.16	1
Pied Avocet *Recurvirostra avosetta*	W	S	0.0028	0.0313	0.5	0.5	0.3	43.33	1.71	1
Northern Lapwing *Vanellus vanellus*	W	S	0.0074	0.0234	0.5	0.2	0.3	33.33	1.54	1
Grey-headed Lapwing *Vanellus cinereus*	STFW	S	0.0274	0.0277 3	0.1	0.5	0.6	40.00	2.76	1
Pacific Golden-Plover *Pluvialis fulva*	W	P	0.0001	0.0132	0.5	0	0.3	26.67	0.66	1
Little Ringed Plover *Charadrius dubius*	W	S	0.0002	0.0036	0.1	0	0.3	13.33	0.19	1
Kentish Plover *Charadrius alexandrinus*	W	S	0.0001	0.0056	0.5	0	0.3	26.67	0.29	1
Common Snipe *Gallinago gallinago*	W	P	0.0002	0.0139	0.5	0	0.3	26.67	0.71	1
Eurasian Curlew *Numenius arquata*	W	P	0.0001	0.0584	0.5	0	0.3	26.67	2.93	1
Eastern Curlew *Numenius madagascariensis*	W	P	0.0001	0.0977	0.5	0	0.3	26.67	4.89	1
Common Redshank *Tringa totanus*	W	S	0.0021	0.0134	0.5	0.2	0.3	33.33	0.77	1
Marsh Sandpiper *Tringa stagnatilis*	W	P	0.0012	0.0084	0.5	0.2	0.3	33.33	0.48	1
Common Green-shank *Tringa nebularia*	W	P	0.0009	0.0252	0.5	0.2	0.3	33.33	1.31	1
Green Sandpiper *Tringa ochropus*	W	P	0.0041	0.0086	0.5	0.5	0.3	43.33	0.64	1
Wood Sandpiper *Tringa glareola*	W	P	0.0002	0.0065	0.5	0	0.3	26.67	0.34	1
Common Sandpiper *Actitis hypoleucos*	W	S	0.0007	0.0053	0.5	0.2	0.3	33.33	0.30	1
Temminck's Stint *Calidris temminckii*	W	P	0.0002	0.0027	0.1	0	0.3	13.33	0.15	1
Brown-headed Gull	W	S	0.0008	0.0665	0.5	0.2	0.3	33.33	3.37	1

Larus brunnicephalus										
Black-headed Gull *Larus ridibundus*	W	P	0.0001	0.0307	0.5	0	0.3	26.67	1.54	1
Relict Gull *Larus relictus*	W	S	0.0022	0.0244	0.5	0.2	0.3	33.33	1.33	1
Siberain Gull *Larus smithsonianus*	W	P	0.0005	0.1371	0.5	0.2	0.3	33.33	6.88	1
Gull-billed Tern *Gelochelidon nilotica*	W	S	0.0001	0.0241	0.5	0	0.3	26.67	1.21	1
Common Tern *Sterna hirundo*	W	S	0.0071	0.0117	0.5	0.5	0.3	43.33	0.94	1
Whiskered Tern *Chlidonias hybridus*	W	S	0.0001	0.0095	0.5	0	0.3	26.67	0.48	1
White-winged Tern *Chlidonias leucopterus*	W	S	0.0041	0.0075	0.5	0.2	0.3	33.33	0.58	1
Great Cormorant *Phalacrocorax carbo*	W	S	0.0004	0.2184	0.5	0	0.3	26.67	10.94	2
White Spoonbill *Platalea leucorodia*	W	S	0.0079	0.2147	0.5	0.5	0.3	43.33	11.13	3
Chinese Pond-Heron *Ardeola bacchus*	W	S	0.0001	0.0388	0.5	0	0.3	26.67	1.95	1
Grey Heron *Ardea cinerea*	W	S	0.0037	0.1456	0.5	0.2	0.3	33.33	7.46	2
Steppe Eagle *Aquila nipalensis*	S	S	0.0001	0.2455	0.5	0	0.6	36.67	12.28	3
Japanese Sparrow Hawk *Accipiter gularis*	T	R	0.0002	0.0097	0.5	0	0.3	26.67	0.50	1
Eurasian Sparrow Hawk *Accipiter nisus*	F	R	0.0002	0.0158	0.5	0	0.3	26.67	0.80	1
Hen Harrier *Circus cyaneus*	S	S	0.0001	0.0479	0.5	0	0.3	26.67	2.40	1
Upland Buzzard *Buteo hemilasius*	S	R	0.0002	0.1642	0.5	0	0.3	26.67	8.22	1
Eastern Buzzard *Buteo buteo*	F	S	0.0001	0.0842	1.0	0	0.3	43.33	4.22	1
Eurasian Eagle-owl *Bubo bubo*	T	R	0.0002	0.1599	0.5	0	0.3	26.67	8.01	1

Little Owl *Athene noctua*	T	R	0.0002	0.0147	0.5	0	0.6	36.67	0.75	1
Long-eared Owl *Asio otus*	T	S	0.0004	0.0270	0.5	0	0.3	26.67	1.37	1
Eurasian Hoopoe *Upupa epops*	A	S	0.0055	0.0071	0.5	0.2	0.9	53.33	0.63	2
Great Spotted Woodpecker *Picoides major*	STFN	R	0.0100	0.0075	0.5	0.2	0.6	43.33	0.88	1
Grey-headed Woodpecker *Picus canus*	STFN	R	0.0033	0.0147	0.5	0.2	0.9	53.33	0.90	2
Common Kestrel *Falco tinnunculus*	A	R	0.0098	0.0217	1.0	0.2	0.9	70.00	1.58	2
Red-footed Falcon *Falco amurensis*	A	S	0.0038	0.0144	0.5	0.2	0.9	53.33	0.91	1
Merlin *Falco columbarius*	A	P	0.0019	0.0193	1.0	0.2	0.9	70.00	1.06	2
Eurasian Hobby *Falco subbuteo*	A	S	0.0118	0.0180	1.0	0.2	0.9	70.00	1.49	2
Red-tailed Shrike *Lanius cristatus*	TS	R	0.0014	0.0032	0.5	0	0.9	46.67	0.23	1
Great Gray Shrike *Lanius excubitor*	TF	W	0.0005	0.0058	0.5	0	0.3	26.67	0.32	1
Chinese Gray Shrike *Lanius sphenocercus*	STFW	S	0.0026	0.0101	0.5	0.2	0.9	53.33	0.64	2
Common Magpie *Pica pica*	A	R	0.4124	0.0240	1.0	0.5	0.9	80.00	21.82	3
Red-billed Chough *Pyrrhocorax pyrrhocorax*	S	R	0.0017	0.0312	0.5	0.2	0.6	43.33	1.64	1
Daurian Jackdaw *Corvus dauuricus*	STWF	R	0.0024	0.0224	1.0	0.2	0.3	50.00	1.24	2
Rook *Corvus frugilegus*	S	R	0.0001	0.0466	1.0	0	0.3	43.33	2.34	1
Carrion Crow *Corvus corone*	SW	R	0.0110	0.0532	1.0	0.5	0.6	70.00	3.21	2
Great Tit *Parus major*	ST	R	0.0031	0.0014	0.1	0.2	0.6	30.00	0.23	1
Mongolian Lark *Melanocorypha monglica*	S	R	0.0001	0.0062	1.0	0	0.9	63.33	0.32	2

Short-toed Lark *Calandrella brachydactyla*	STWF	S	0.0105	0.0029	1.0	0.2	0.9	70.00	0.67	2
Asian Short-toed Lark *Calandrella cheleensis*	ST	R	0.0098	0.0028	1.0	0.2	0.9	70.00	0.63	2
Crested Lark *Galerida cristata*	A	R	0.1118	0.0044	0.5	0.2	0.9	53.33	5.81	2
Eurasian Skylark *Alauda arvensis*	STN	S	0.0017	0.0036	1.0	0.2	0.3	50.00	0.26	2
Bank Swallow *Riparia riparia*	STWN	S	0.0483	0.0015	1.0	1	0.9	96.67	2.49	2
Barn Swallow *Hirundo rustica*	A	S	0.0791	0.0019	1.0	0.5	0.9	80.00	4.05	2
Yellow-rumoed Willow Warbler *Phylloscopus proregulus*	T	S	0.0002	0.0005	0.5	0	0.3	26.67	0.04	1
Yellow-browed Warbler *Phylloscopus inornatus*	ST	P	0.0004	0.0008	0.5	0.2	0.6	43.33	0.06	1
Long-tailed Tit *Aegithalos caudatus*	ST	R	0.0013	0.0009	0.5	0.2	0.6	43.33	0.11	1
White-cheeked Starling *Sturnus cineraceus*	A	S	0.0590	0.0089	0.5	1	0.6	70.00	3.40	2
Red-necked Thrush *Turdus ruficollis*	SW	P	0.0011	0.0096	0.5	0.2	0.6	43.33	0.53	1
Dusky Thrush *Turdus eunomus*	T	P	0.0002	0.0081	0.5	0	0.3	26.67	0.42	1
Siberian Rubythroat *Luscinia calliope*	S	P	0.0001	0.0023	0.5	0	0.9	46.67	0.12	1
Red-flanked Bluetail *Tarsiger cyanurus*	S	S	0.0001	0.0014	0.5	0	0.3	26.67	0.08	1
Isabelline Wheatear *Oenanthe isabellina*	T	R	0.0001	0.0028	0.5	0	0.6	36.67	0.15	1
Wheatear *Oenanthe oenanthe*	T	S	0.0002	0.0026	0.1	0	0.6	23.33	0.14	1
Pied Wheatear *Oenanthe*	STF	S	0.0039	0.0018	0.1	0.2	0.9	40.00	0.28	1

pleschanka										
Desert deserti *Oenanthe deserti*	S	R	0.0001	0.0024	0.5	0	0.6	36.67	0.12	1
Taiga Flycatcher *Ficedula parva*	T	P	0.0005	0.0012	0.5	0.2	0.6	43.33	0.08	1
Siberian Accentor *Prunella montanella*	S	W	0.0001	0.0016	0.5	0	0.3	26.67	0.09	1
Tree Sparrow *Passer montanus*	A	R	1.0000	0.0021	1.0	1	0.9	96.67	50.11	3
Yellow Wagtail *Motacilla flava*	W	S	0.0001	0.0020	0.1	0	0.3	13.33	0.11	1
Yellow-headed Wagtail *Motacilla citreola*	WN	S	0.0013	0.0023	0.5	0.2	0.6	43.33	0.18	1
Gray Wagtail *Motacilla cinerea*	W	S	0.0018	0.0019	0.5	0.2	0.3	33.33	0.18	1
White Wagtail *Motacilla alba*	A	S	0.0050	0.0024	0.5	0.2	0.9	53.33	0.37	1
Olive-backed Pipit *Anthus hodgsoni*	T	S	0.0001	0.0024	0.5	0	0.6	36.67	0.13	1
Common Rosefinch *Carpodacus erythrinus*	T	P	0.0001	0.0024	0.5	0	0.3	26.67	0.12	1
Greenfinch *Carduelis sinica*	STFN	R	0.0225	0.0019	0.5	0.5	0.6	53.33	1.22	2
Meadow Bunting *Emberiza cioides*	ST	R	0.0008	0.0021	0.5	0.2	0.6	43.33	0.15	1
Little Bunting *Emberiza pusilla*	S	W	0.0005	0.0015	0.5	0.2	0.6	43.33	0.10	1
Ochre-rumped Bunting *Emberiza yessoensis*	ST	S	0.0006	0.0014	0.5	0.2	0.6	43.33	0.10	1

Habitat distribution：S: Shrubland, T: Woodland, F: Farmland, N: Neighborhood, W: Wetlands, A: Were distributed in various habitats. Resident type: R: Resident species, P: Migrants species, S: Summer visitors species, W: Winter visitors.
